# Identification and validation of key genes associated with atrial fibrillation in the elderly

**DOI:** 10.3389/fcvm.2023.1118686

**Published:** 2023-03-29

**Authors:** Chuanbin Liu, Jing Zeng, Jin Wu, Jing Wang, Xin Wang, Minghui Yao, Minghua Zhang, Jiao Fan

**Affiliations:** ^1^ Western Medical Branch of PLA General Hospital, Beijing, China; ^2^ Department of Endocrinology, The Second Medical Centre & National Clinical Research Centre for Geriatric Disease, Chinese PLA General Hospital, Beijing, China; ^3^ Tianjin Key Laboratory of Risk Assessment and Control Technology for Environment and Food Safety, Institute of Environmental and Operational Medicine, Tianjin, China; ^4^ Department of General Medicine, The First Medical Center of PLA General Hospital, Beijing, China; ^5^ Department of Ophthalmology, PLA Strategic Support Force Characteristic Medical Center, Beijing, China; ^6^ Department of Cardiovascular Surgery, the First Medical Center of PLA General Hospital, Beijing, China; ^7^ Clinical Pharmacy Laboratory, Chinese PLA General Hospital, Beijing, China; ^8^ Institute of Geriatrics, The Second Medical Centre & National Clinical Research Centre for Geriatric Disease, Chinese PLA General Hospital, Beijing, China

**Keywords:** atrial fibrillation, hub genes, weighted gene co-expression network analysis, elderly, biomarkers

## Abstract

**Background:**

Atrial fibrillation (AF) is the most common cardiac arrhythmia and significantly increases the risk of stroke and heart failure (HF), contributing to a higher mortality rate. Increasing age is a major risk factor for AF; however, the mechanisms of how aging contributes to the occurrence and progression of AF remain unclear. This study conducted weighted gene co-expression network analysis (WGCNA) to identify key modules and hub genes and determine their potential associations with aging-related AF.

**Materials and methods:**

WGCNA was performed using the AF dataset GSE2240 obtained from the Gene Expression Omnibus, which contained data from atrial myocardium in cardiac patients with permanent AF or sinus rhythm (SR). Hub genes were identified in clinical samples. Gene Ontology (GO) and Kyoto Encyclopedia of Genes and Genomes (KEGG) enrichment analyses were also performed.

**Results:**

Green and pink were the most critical modules associated with AF, from which nine hub genes, *PTGDS, COLQ, ASTN2, VASH1, RCAN1, AMIGO2, RBP1, MFAP4,* and *ALDH1A1*, were hypothesized to play key roles in the AF pathophysiology in elderly and seven of them have high diagnostic value. Functional enrichment analysis demonstrated that the green module was associated with the calcium, cyclic adenosine monophosphate (cAMP), and peroxisome proliferator-activated receptors (PPAR) signaling pathways, and the pink module may be associated with the transforming growth factor beta (TGF-*β*) signaling pathway in myocardial fibrosis.

**Conclusion:**

We identified nine genes that may play crucial roles in the pathophysiological mechanism of aging-related AF, among which six genes were associated with AF for the first time. This study provided novel insights into the impact of aging on the occurrence and progression of AF, and identified biomarkers and potential therapeutic targets for AF.

## Introduction

Atrial fibrillation (AF) is the most common cardiac arrhythmia and increases the risk of myocardial infarction (MI), heart failure (HF), and stroke, contributing to a higher mortality rate (1). Increasing age is a prominent risk factor for AF, and approximately 70% of individuals with AF are between the ages of 65 and 85 years (2, 3). One in three individuals of European descent over the age of 55 has AF (4). With the aging population, the prevalence of AF and the absolute number of AF patients will continuously increase worldwide in the coming decades. With the continuous development and promotion of new technologies, such as radiofrequency ablation (RA) surgery and left atrial appendage closure (LAAC), treatment options for AF have evolved considerably. However, the mechanisms of how aging contributes to the development and progression of AF are still unclear. Aggressive antithrombotic therapy effectively prevents AF complications, but the low awareness of AF results in some patients not receiving timely and effective treatment, eventually causing strokes or death (5). In addition, paroxysmal and asymptomatic AF cannot be easily diagnosed using electrocardiography (ECG) and Holter monitoring (6). Thus, biomarkers for AF screening are urgently necessary.

AF is accompanied by a complex biological process under the synergistic action of multiple genes (7, 8). The traditional biological methods to study the expression and function of many genes cannot reveal a more comprehensive systemic behavior through gene interactions. Genetics plays an important role in studying the etiology of many complex diseases. Individual genes do not work alone but interact with other genes to influence human health. Studies have shown that each gene interacts with an average of 4–8 other genes and is involved in ten biological functions (9). Gene networks can predict the function of new genes and identify hundreds of genes associated with complex diseases, thus, predicting relevant targets for therapeutic intervention in diseases. Weighted gene co-expression network analysis (WGCNA) is a commonly used method for constructing gene networks, detecting gene modules, and identifying hub genes in modules (10). The WGCNA has been widely used to identify key genes involved in human disease progressions, such as cancer, Alzheimer's disease (AD), and mental disorders. Moreover, WGCNA has been validated as a valuable method to identify the underlying mechanisms, potential biomarkers, or therapeutic targets by focusing on key modules (11–13). Previous studies on the mechanisms of AF have concentrated on electrical and structural remodeling, with relatively few identifying comprehensive regulatory networks (14, 15). Li et al. (16) screened several AF-related genes and pathways using the WGCNA method and performed preliminary validation at the animal level. In contrast, this study focused on the differences in the expression of key genes between elderly patients with AF and non-AF patients.

The present study used the GSE2240 dataset downloaded from the Gene Expression Omnibus (GEO) database to perform WGCNA to identify the AF closely associated gene modules for further analysis of Gene Ontology (GO) and Kyoto Encyclopedia of Genes and Genomes (KEGG). Hub genes in the modules highly associated with AF were identified, and the biological functions and pathways of genes in the key modules were analyzed. Quantitative reverse transcription polymerase chain reaction (RT-qPCR) was used to detect peripheral blood leukocytes in elderly AF patients and controls to verify the results of the hub genes in critical modules. This study revealed the potential regulatory mechanisms underlying aging in AF and identified novel biomarkers and therapeutic targets.

## Materials and methods

### Dataset information

The AF dataset, GSE2240, was obtained from the National Center for Biotechnology Information (NCBI) Gene Expression Omnibus (GEO; https://www.ncbi.nlm.nih.gov/geo/). GSE2240 contained data on 30 right atrial appendages from 10 patients with permanent AF, defined as AF duration longer than 3 months, and 20 patients with sinus rhythm (SR), with no history of AF.

### Data pre-processing

Raw data were pre-processed identically R for background correction and normalization. R package annotation was conducted to match probes and gene symbols, and the probes matching several genes were removed. The median was regarded as the final expression value for the gene matched by multiple probes. We calculated the standard deviation (SD) for each gene and ranked them from the largest to the smallest, and the top 5000 genes were chosen for WGCNA refer to the official tutorials.

### Construction of the weighted gene co-expression network

A gene co-expression network was constructed using the R package WGCNA (10, 17). In this analysis, nodes represented genes, and edges represented the degree of co-expression. Firstly, the hclust function was used to cluster the samples to determine whether there were outliers samples. Then, weighted correlation coefficients were introduced to calculate the adjacency matrix of the expression profile genes. The co-expression similarity between genes *i* and *j* was defined as *S_ij_* = |cor(*i*,*j*)|. The correlated adjacency of the genes was further analyzed using the power function: *a_ij_* = |*S_ij_*|*^β^*. After that, soft threshold *β* was chosen within a specific range to satisfy the scale-free network and better network connectivity. Fit index *R*^2^ > 0.85 was set to make the connections between genes obey the approximate scale-free network distribution. And the pickSoftThreshold function was applied to automatically filter the appropriate soft threshold *β*. Finally, the blockwiseModules function was used for network construction and module detection to generate the topological overlap matrix (TOM) with a minimum module size of 50 and a merge cut height of 0.25 for co-expressed gene modules. Based on the heterogeneity of TOM, the genes with similar expression patterns were divided into the same module by means of average link hierarchical clustering, and the gene modules were identified by dynamic shearing tree method.

### Correlation analysis of co-expression modules with clinical traits

Gene modules are clusters of genes that are closely related to co-expression. WGCNA uses a hierarchical clustering approach to identify gene modules and represents each cluster with a different color. Genes not assigned to any module are placed in gray modules. Principal component analysis was performed for each module, and the module eigengenes (MEs) were calculated using the first principal component representing the overall expression level of the module. Module-trait correlation coefficients were calculated to provide a heat map of the correlation coefficients between modules and traits (18). Then, we screened the gene modules significantly associated with the traits using the eigenvectors of modules, correlation coefficients of traits, and module significance (*P *< 0.05). The values of gene significance (GS) and module membership (MM) were calculated. GS represents the correlation between gene expression and traits within the calculated module, and MM represents the correlation coefficient between the expression of a gene and the expression of the main component of the gene within the module. Finally, the list of genes calculated by the networkScreening function was screened by setting the value range of GS, MM, and q.weighted to identify and characterize the key hub genes.

### Network visualization and functional enrichment analysis

Gene modules significantly associated with traits were selected, and network maps were drawn by weighted co-expression relationships between genes using the Cytoscape software. Functional annotation analysis of genes in the core module based on the Gene Ontology (GO) database and signaling pathway enrichment analysis of genes in the core module based on the Kyoto Encyclopedia of Genes and Genomes (KEGG) database were performed to determine the biological functions and potential biological pathways of genes in the trait-related modules.

### Validation of the clinically related genes by RT-qPCR

All protocols and the use of human blood samples were in accordance with the Declaration of Helsinki and approved by the Human Ethics Review Committee of the Chinese PLA General Hospital. All leukocyte samples were obtained from the PLA General Hospital Biosample Bank, including 9 elderly AF patients and 9 elderly non-AF controls. Blood samples were collected in EDTA anticoagulation tubes. Leukocytes were isolated from human peripheral blood by Ficoll density gradient centrifugation according to the manufacturer's instructions and stored at −80°C until further analysis. Detailed patient characteristics are summarized in Supplementary Table S1.

**Figure 1:**
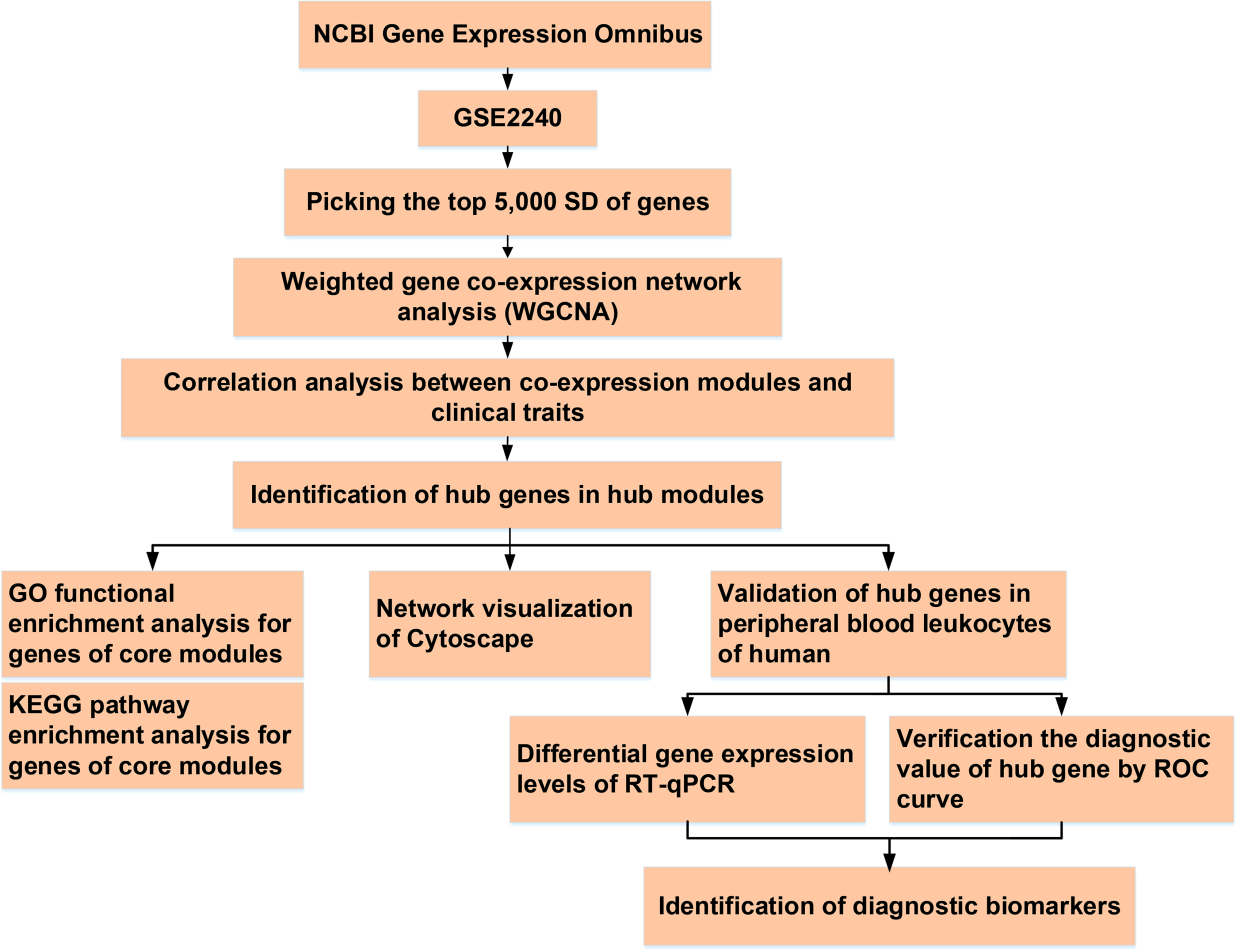
The flowchart of analysis process.

Total RNA was extracted from leukocytes using an RNAprep Pure Blood Kit (TIANGEN Biotech Corporation, China), according to the standard protocol. Absorption spectrophotometry using NanoDrop-1,000 (Thermo Fisher Scientific, Yokohama, Japan) was used to determine RNA concentrations and purity (260/280 ratio >1.8). The RNA (1 *μ*g per sample) was reverse transcribed using the High-Capacity cDNA Reverse Transcription Kit (Applied Biosystems, CA, USA) in a total reaction volume of 20 *μ*l, following the manufacturer's instructions. Then, RT-qPCR was performed as follows: pre-denatured at 95°C for 30 s; denatured at 95°C for 5 s, and 40 cycles at 60°C for 30–34 s. All experiments were performed in triplicates. The housekeeping gene GAPDH was used as the endogenous reference. The primer sequences of *PTGDS, COLQ, ASTN2, VASH1, RCAN1, AMIGO2, RBP1, MFAP4,* and *ALDH1A1* used in this study are listed in Supplementary Table S2. The relative mRNA expression levels were calculated using the 2^−^*^ΔΔ^*^Ct^ method. A flow chart of this study is shown in Figure 1.

### Statistical analysis

Statistical analyses were performed using SPSS statistical package (version 21.0; SPSS Inc., Chicago, USA). Continuous variables were presented as mean ± standard error (SE), and categorical variables were expressed as percentages. Student's *t*-test or the chi-square test was used to determine the differences between AF patients and controls. The sample size was analyzed by PASS (version 15.0; NCSS, USA). Differences were considered statistically significant at *P* < 0.05.

## Results

### Construction of WGCNA network

**Figure 2:**
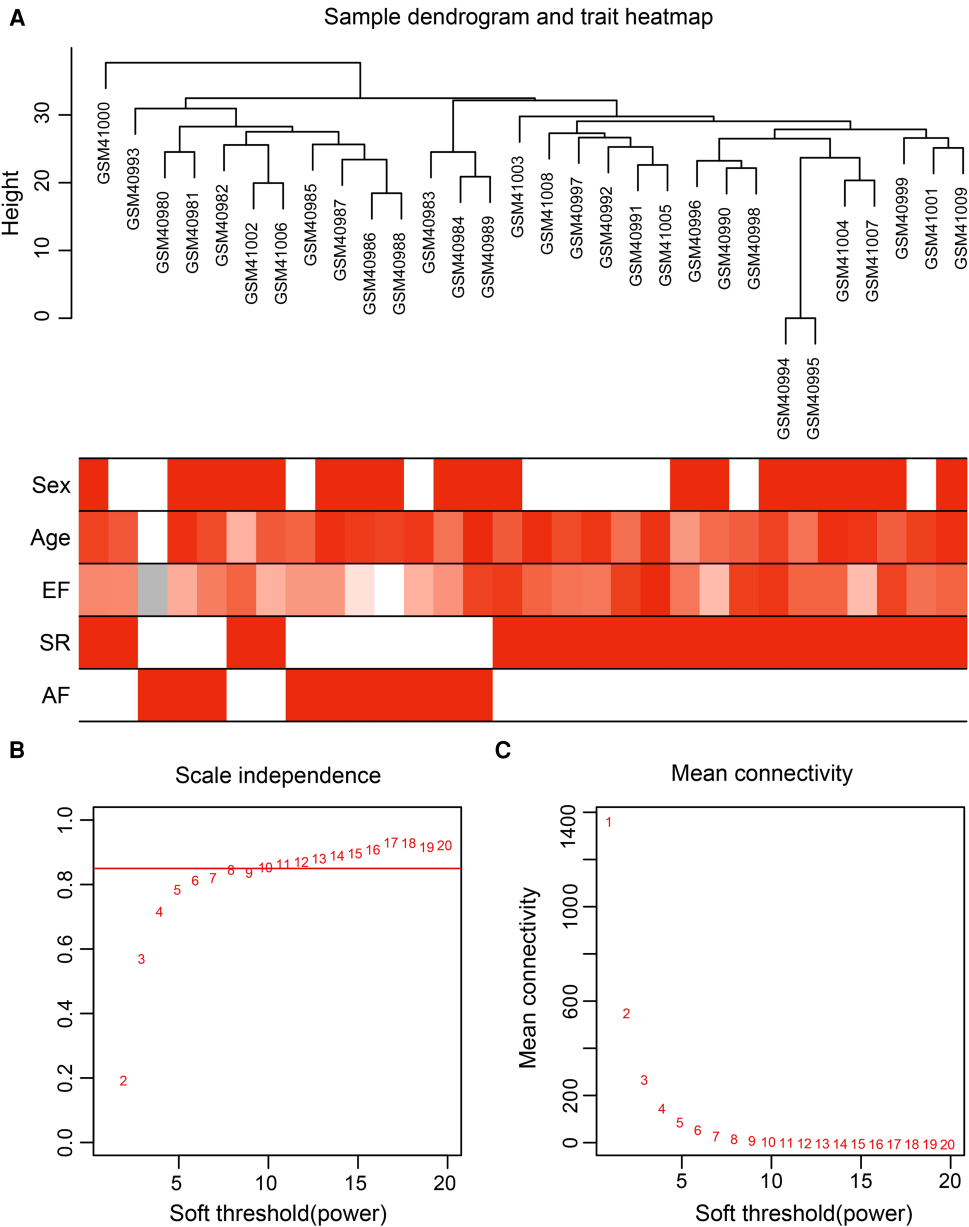
Data preparation of co-expression network. (**A**) Clinical feature heatmap and hierarchical clustering dendrogram. The degree of heatmap from white to dark red recognizes low to high levels of clinical characteristics, and gray indicates unavailable data. The clinical feature includes sex, age, ejection fraction (EF), SR and AF. (**B,C**) A scale-free co-expression network estimated by the soft-thresholding powers, and the best power value *β* was obtained as 8.

**Figure 3:**
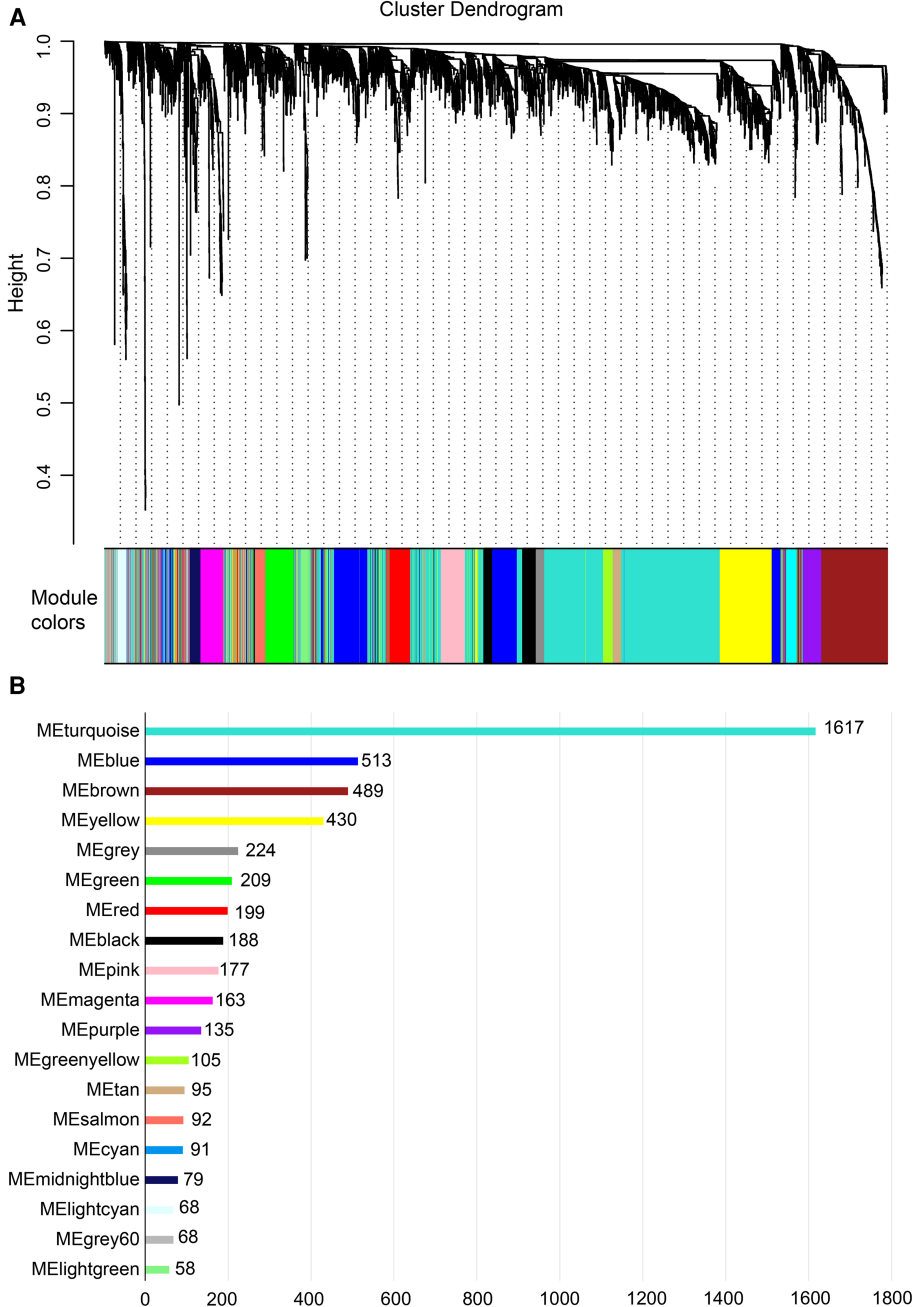
Construction of co-expression network. (**A**) Nineteen co-expression modules constructed by clustering dendrograms and partitioned into different module colors (non-clustering genes shown in gray). (**B**) The bar plot of numbers in modules.

Gene expression values of all samples were subjected to sample clustering and phenotypic heat map analysis. As shown in Figure 2A, there were no significant outlier samples. Therefore, all the samples were included in the subsequent data analysis. Using the WGCNA, we calculated and selected *β* = 8 as the soft threshold for the dataset based on the scale-free network fit index and average connectivity (Figures 2B,C). The adjacency and TOM matrices between genes were calculated. A hierarchical clustering tree of the genes was constructed based on the TOM matrix. Then, the genes were divided into 19 modules using the dynamic shearing tree method. Each module was represented by a colored rectangle, and the vertical coordinates were the gene occupancy ratio. The tree branches correspond to 10 different gene modules, each leaf on the tree corresponds to a gene, and similar genes are clustered into modules of the same color (Figures 3A,B). The module name and the number of genes included in each module were as follows: turquoise (1,617), blue (513), brown (489), yellow (430), green-yellow (105), black (188), magenta (163), pink (177), red (199), purple (135), yellow (332), grey (21), tan (95), salmon (92), cyan (91), midnight blue (79), light cyan (68), grey60 (68), and light green (58). Genes from each module are listed in Supplementary Table S3. The genes in the gray module could not be clustered to any other module and were removed from the subsequent analysis.

### Correlation between modules and clinical traits

**Figure 4:**
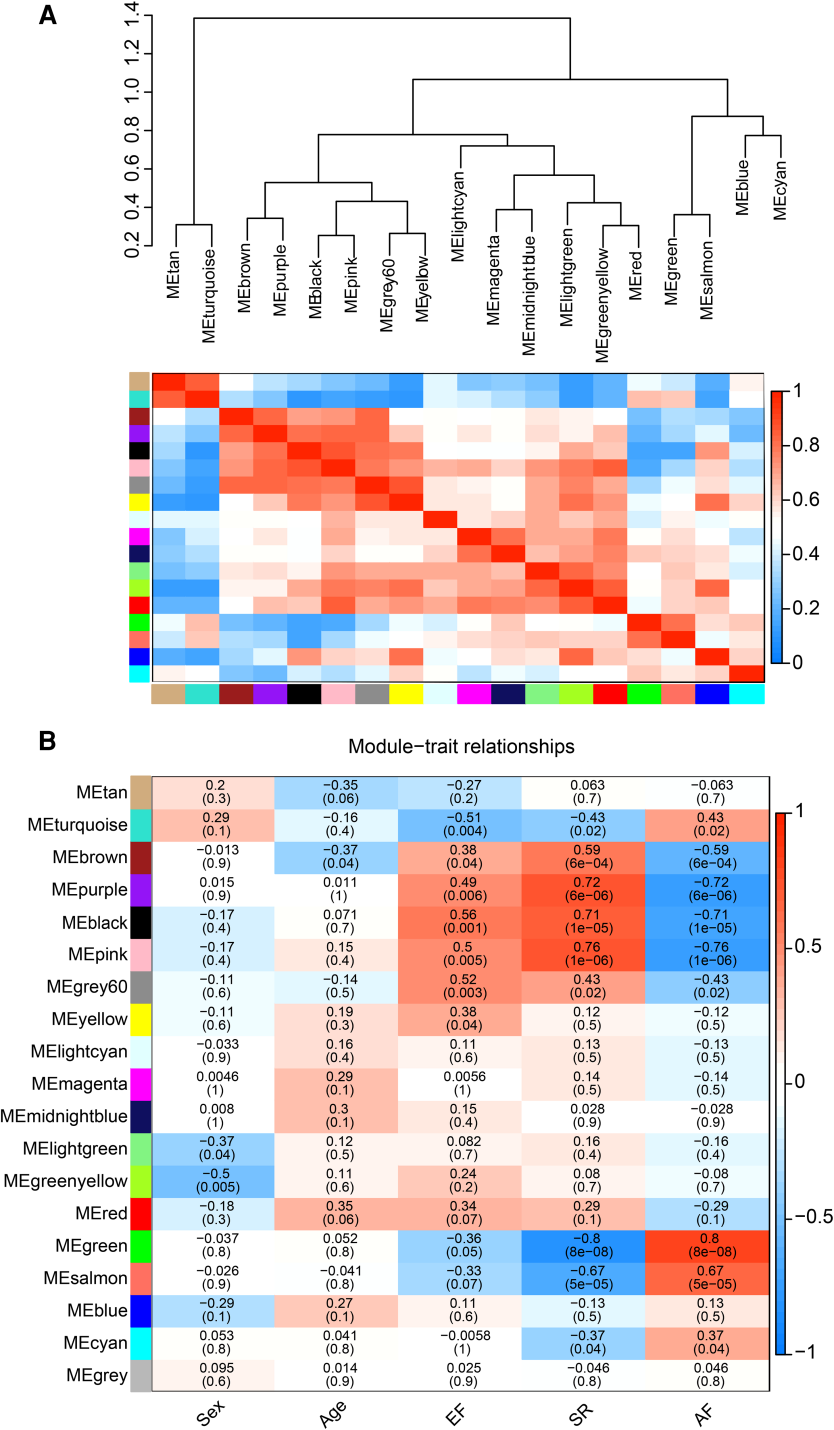
Correlation between co-expression modules and clinical traits. (**A**) Hierarchical clustering trees and heatmap of different modules. The red shows positive correlation and blue shows negative correlation. (**B**) Correlation relationship between each network module and traits. The values in the matrix cell indicate the correlation coefficient and the related *p*-value.

The correlation heat map between co-expression modules and clinical traits was drawn by calculating the relationship between each gene module and clinical traits. Hierarchical clustering and heat map analyses were performed for each module. The correlations between the modules are shown in Figure 4A. The pink and green modules are distributed in different clustering subtrees. In this study, the pink module exhibited the highest positive correlation (*r *= 0.76, *P *< 0.0001) with the SR phenotype, and the green module exhibited the highest negative correlation with the AF phenotype (*r* = −0.8, *P *< 0.0001) (Figure 4B). Therefore, we focused on these two modules in the subsequent analysis.

**Figure 5:**
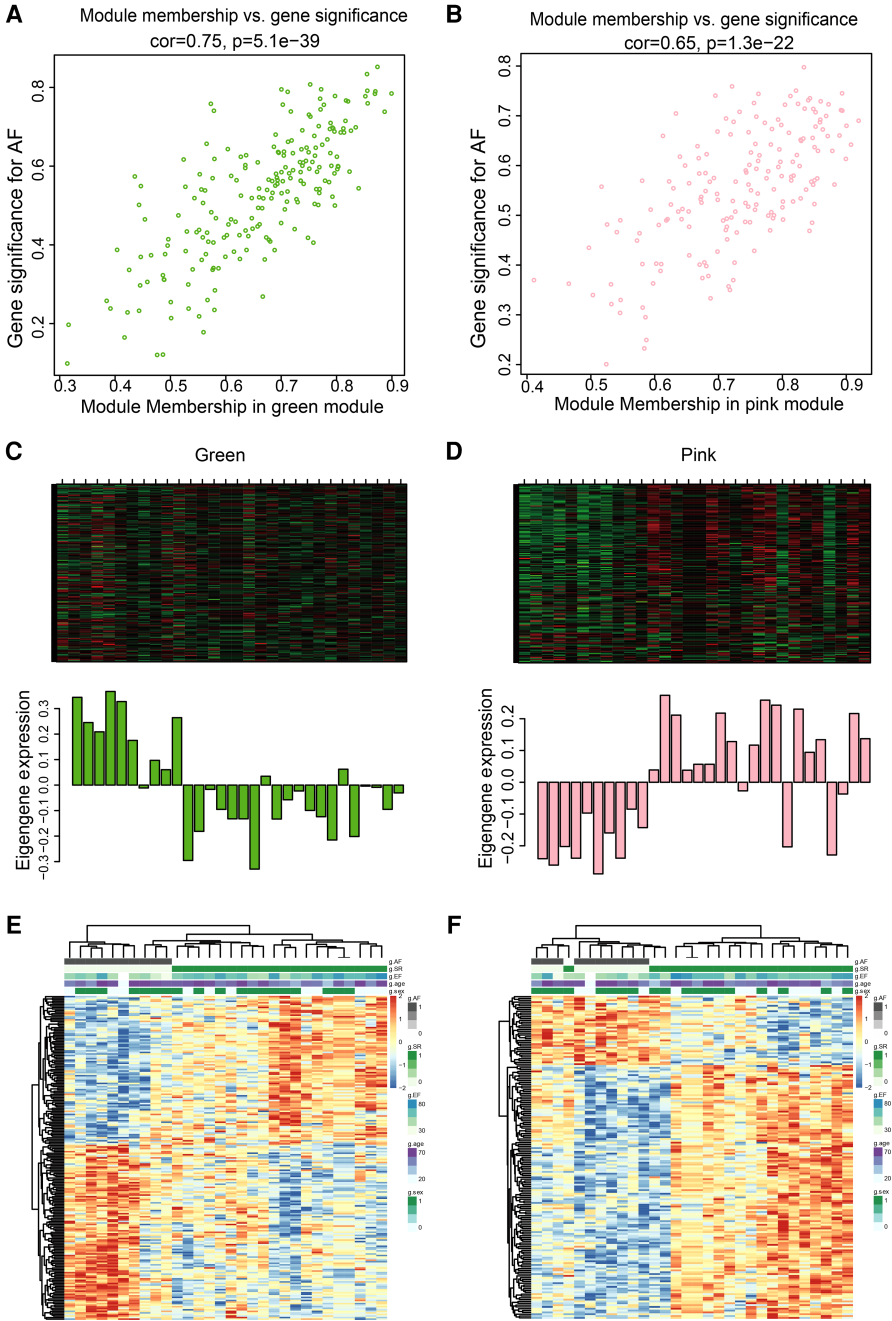
Correlation between green and pink modules with SR and AF. (**A,B**) Scatter plot in green (**A**) and pink (**B**) modules with GS (*y*-axis) and MM (*x*-axis). (**C,D**) The expression level of genes in green (**C**) and pink (**D**) modules. In the heatmap, green indicates the low expression and red indicates the high expression for samples. (**E,F**) Heatmap and clustering map of clinical traits in green (**E**) and pink (**F**) modules. In the clustering map, the varying shades of color recognizes low to high levels of expression in each clinical trait.

The pink and green modules were used as key modules for GS and MM analyses, respectively. The correlation coefficient between GS and MM was *r* = 0.75, *P* < 0.0001 (Figure 5A) and *r* = 0.65, *P* < 0.0001 (Figure 5B) for the green and pink modules, respectively. Figures 5C,D represent the heat map of eigengene expression for the two modules. Figures 5E,F show the heat and clustering map between the genes of the two modules and each clinical trait.

Finally, three criteria were used to screen for key hub genes in the pink and green modules: GS > 0.65, MM > 0.8, and q.weighted <0.01. After excluding non-coding genes, 16 hub genes were screened in the green module (Table 1) and 23 hub genes in the pink module (Table 2).

**Table 1:** Hub genes screened in the green module.

Gene	GS	q.Weighted	*p*.Weighted
*SFRP5*	0.76	6.17 × 10^–7^	3.27 × 10^–9^
*ASTN2*	0.78	3.80 × 10^–7^	1.46 × 10^–9^
*IGFBP2*	0.78	1.14 × 10^–6^	1.03 × 10^–8^
*GPR22*	0.83	6.15 × 10^–7^	3.02 × 10^–9^
*PPP1R1A*	0.76	2.00 × 10^–7^	2.99 × 10^–10^
*COLQ*	0.79	2.50 × 10^–7^	5.94 × 10^–10^
*COG5*	0.79	2.00 × 10^–7^	2.98 × 10^–10^
*DDAH1*	0.69	2.50 × 10^–7^	5.87 × 10^–10^
*VASH1*	0.85	1.74 × 10^–5^	4.13 × 10^–7^
*RCAN1*	0.78	2.50 × 10^–7^	6.95 × 10^–10^
*MARCH3*	0.70	6.48 × 10^–6^	1.09 × 10^–7^
*RPS6KA5*	0.69	4.98 × 10^–5^	1.67 × 10^–6^
*DGKD*	0.70	6.65 × 10^–6^	1.14 × 10^–7^
*ART3*	0.74	7.94 × 10^–5^	3.25 × 10^–6^
*C9orf16*	0.71	5.06 × 10^–7^	2.35 × 10^–9^
*PTGDS*	0.69	7.19 × 10^–6^	1.29 × 10^–7^

**Table 2:** Hub genes screened in the pink module.

Gene	GS	q.Weighted	*p*.Weighted
*ALDH1A1*	0.72	3.80 × 10^–7^	1.36 × 10^–9^
*ABCA8*	0.71	2.50 × 10^–7^	6.34 × 10^–10^
*FBLN1*	0.72	1.92 × 10^–5^	4.77 × 10^–7^
*AKAP12*	0.66	3.53 × 10^–5^	1.10 × 10^–6^
*PLP1*	0.75	2.50 × 10^–7^	4.81 × 10^–10^
*C7*	0.66	5.32 × 10^–6^	8.29 × 10^–8^
*MFAP4*	0.71	8.64 × 10^–7^	5.72 × 10^–9^
*RBP1*	0.71	1.72 × 10^–6^	1.95 × 10^–8^
*PPAP2B*	0.70	7.50 × 10^–7^	4.44 × 10^–9^
*EPB41L2*	0.69	1.03 × 10^–6^	8.45 × 10^–9^
*CD34*	0.66	0.000123	5.97 × 10^–6^
*AMIGO2*	0.75	6.47 × 10^–7^	3.59 × 10^–9^
*MCF2L*	0.80	6.17 × 10^–7^	3.29 × 10^–9^
*FAT4*	0.69	9.29 × 10^–7^	6.34 × 10^–9^
*DCLK1*	0.68	5.58 × 10^–6^	9.06 × 10^–8^
*TGFBR2*	0.74	8.23 × 10^–7^	5.27 × 10^–9^
*AMD1*	0.73	5.06 × 10^–7^	2.38 × 10^–9^
*TIAM1*	0.68	8.26 × 10^–6^	1.59 × 10^–7^
*GRIP2*	0.73	7.50 × 10^–7^	4.48 × 10^–9^
*ID2*	0.69	1.60 × 10^–5^	3.65 × 10^–7^
*LPAR1*	0.67	3.48 × 10^–5^	1.07 × 10^–6^
*ZCCHC24*	0.71	1.02 × 10^–6^	8.05 × 10^–9^
*DCN*	0.71	1.60 × 10^–5^	3.70 × 10^–7^
*CACNA2D3*	0.67	1.51 × 10^–5^	3.39 × 10^–7^
*PTTG1*	0.72	7.59 × 10^–6^	1.39 × 10^–7^
*CALHM2*	0.65	0.00028	1.64 × 10^–5^

### Network visualization and functional enrichment analysis

**Figure 6:**
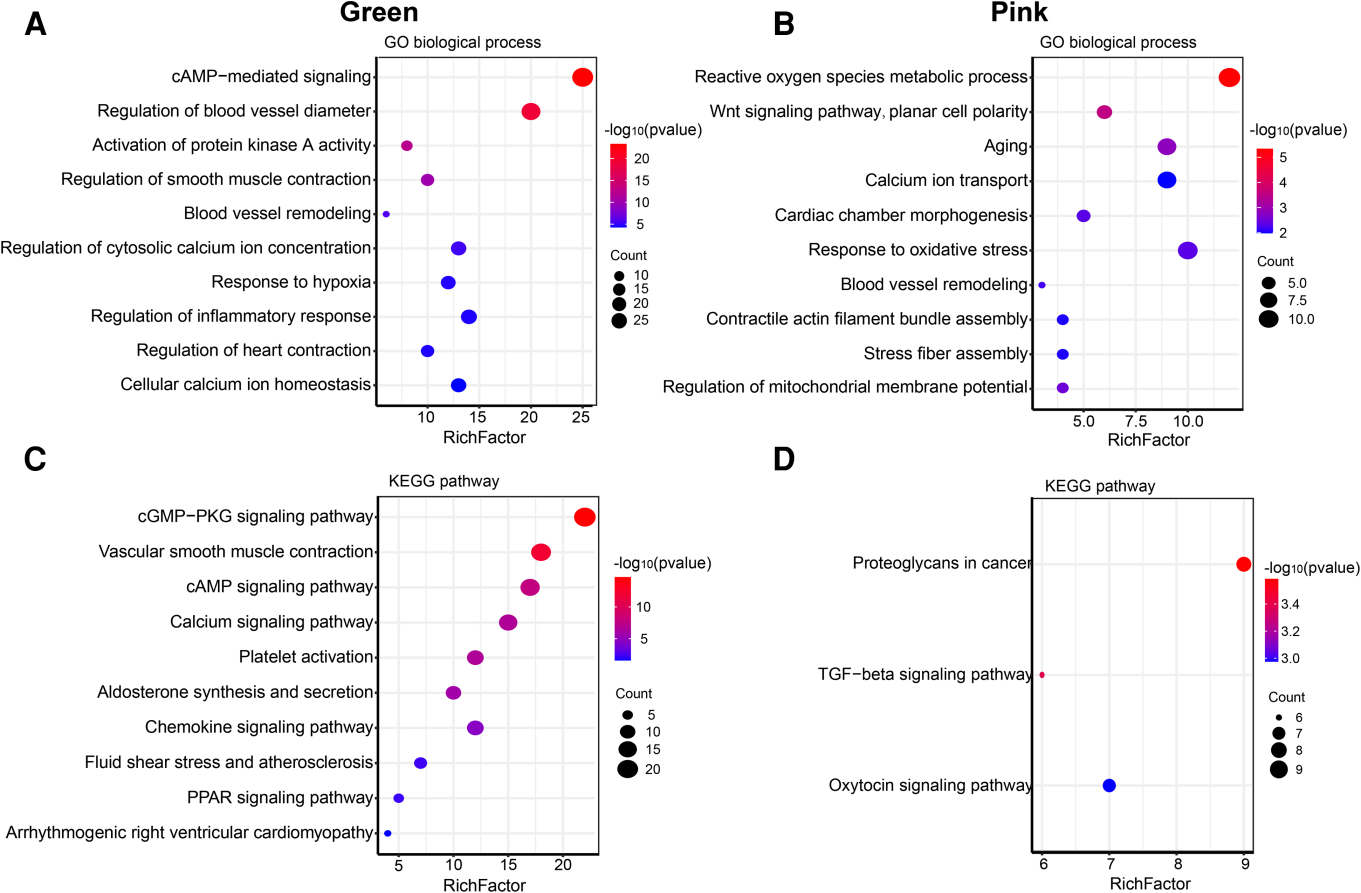
Go and KEGG enrichment analysis of green and pink modules. (**A**) GO analysis of biological process of green module. (**B**) GO analysis of biological process of pink module. (**C**) KEGG pathway analysis of green module. (**D**) KEGG pathway analysis of pink module. The size of the bras represents the number of genes, and the color of the dots represents the −log10(***P***) value, the *x*-axis represents the RichFactor of genes.

The size of the node shape represents the number of edges connected to that node, and the width of the edge represents the weight of the connection between two nodes. GO enrichment analysis was performed on the hub genes in the two modules separately; the respective module genes are shown in Figure 6. The gene functions of the green module were enriched in cyclic adenosine monophosphate (cAMP)-mediated signaling (GO:0019933), activation of protein kinase A activity (GO:0034199), regulation of inflammatory response (GO:0050727), cellular calcium ion homeostasis (GO:0006874), and other biological processes. In addition, the gene functions of the pink module were mainly enriched in the reactive oxygen species (ROS) metabolic process (GO:0072593), Wnt signaling pathway (GO:0060071), aging (GO:0007568), calcium ion transport (GO:0006816), and regulation of mitochondrial membrane potential (GO:0051881).

**Figure 7:**
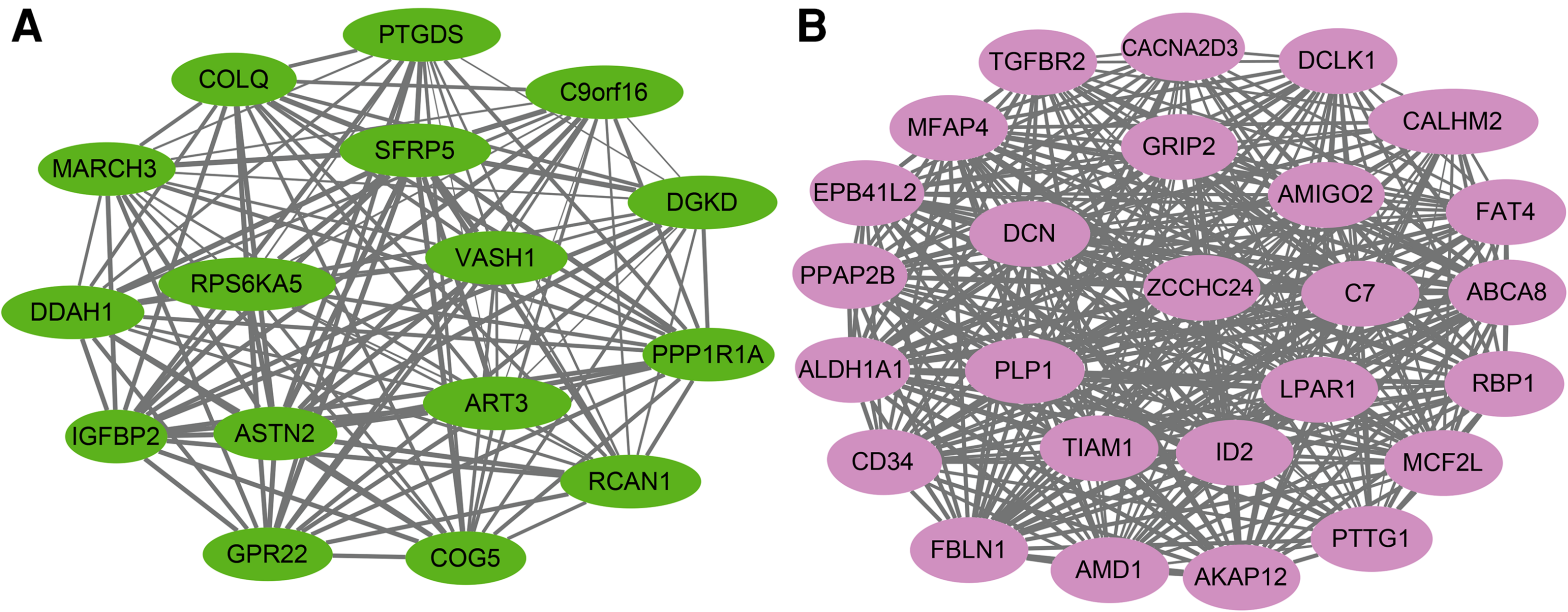
Hub gene map of the green module (**A**) and pink module (**B**).

The KEGG pathway analysis showed that the green module was mainly enriched in the calcium signaling pathway (hsa04020), cAMP signaling pathway (hsa04024), and peroxisome proliferator-activated receptors (PPAR) signaling pathway (hsa03320). The pink module gene was mainly enriched in the transforming growth factor beta (TGF-*β*) signaling pathway (hsa04350). The corresponding data are presented in Supplementary Tables S4, S5. The screened hub gene list was inputted into the Cytoscape software. The interaction relationship graph between the co-expression network genes was made according to the weights of the genes, as shown in Figures 7A,B.

### Validation of hub genes in human peripheral blood leukocytes

**Figure 8:**
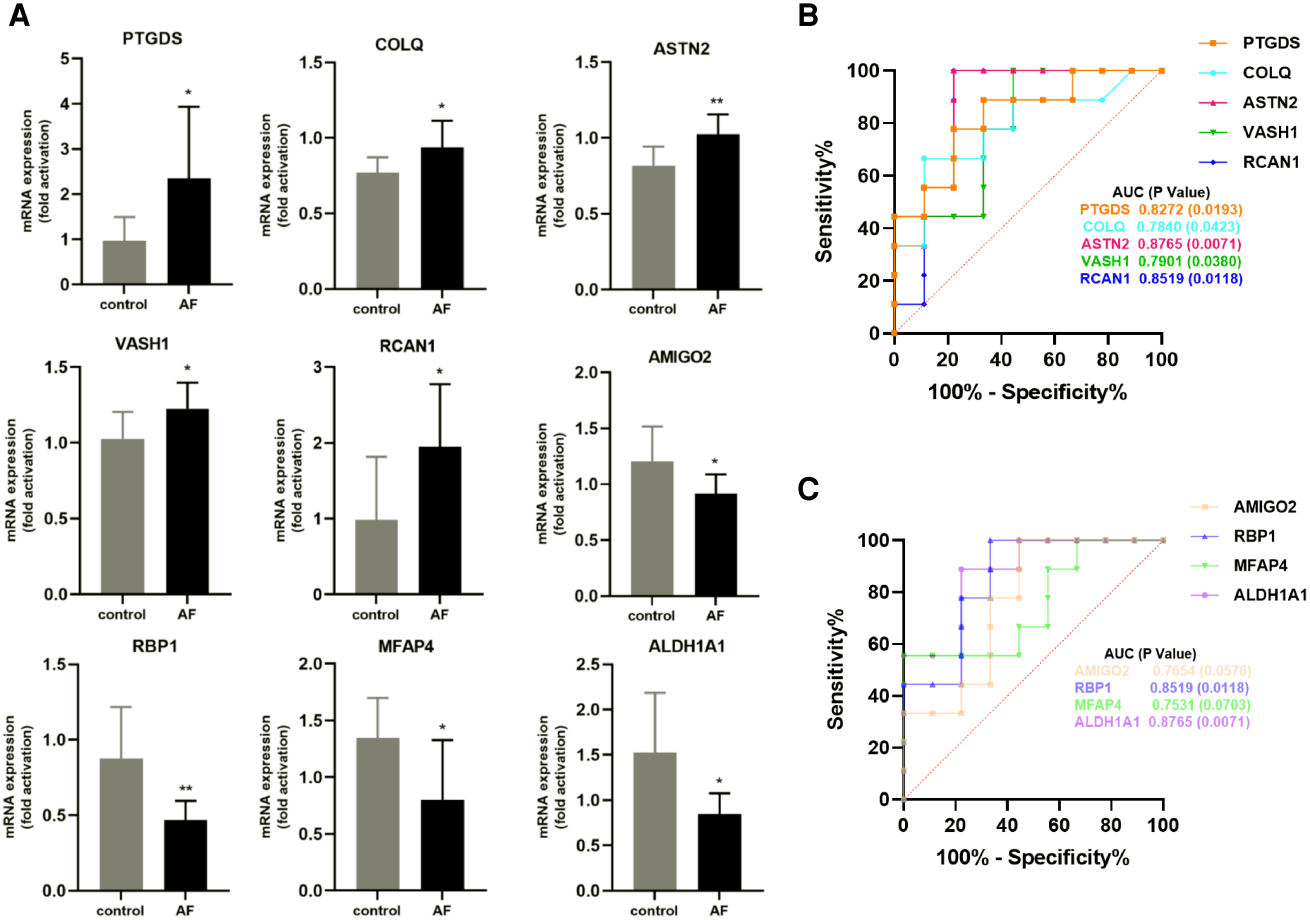
Validation of hub genes in human peripheral blood leukocytes. (**A**) Relative mRNA expression level of nine hub genes in AF patients and controls (* *P* < 0.05, **P < 0.01). (**B**) ROC curve for five hub genes from the green module. (**C**) ROC curve for four hub genes from the pink module.

We performed RT-qPCR to detect gene expression levels in peripheral blood leukocytes collected from elderly AF patients and controls to validate the hub genes further. As shown in Figure 8A, we found that the expression of *PTGDS, COLQ, ASTN2, VASH1*, and *RCAN1* genes from the green module was significantly increased in the AF group compared with those in the control (SR) group. In contrast, the expression of *AMIGO2, RBP1, MFAP4*, and *ALDH1A1* genes in the pink module was decreased in the AF group compared with those in the SR group, suggesting that these genes can potentially be involved in the molecular mechanisms of AF.

Furthermore, based on gene expression levels, receiver operating characteristic (ROC) analysis was used to investigate whether the nine genes could predict AF in the elderly. As shown in Figure 8B, the area under the curve (AUC) and *P* value of *PTGDS, COLQ, ASTN2, VASH1*, and *RCAN1* from the green module were 0.8272 (0.0193), 0.7840 (0.0423), 0.8765 (0.0071), 0.7901 (0.0380), and 0.8519 (0.0118), respectively. The AUC and *P* value of *AMIGO2, RBP1, MFAP4,* and *ALDH1A1* in the pink module was 0.7654 (0.0576), 0.8519 (0.0118), 0.7531 (0.0703), and 0.8765 (0.0071), respectively (Figure 8C). These results indicate that the seven genes *PTGDS, COLQ, ASTN2, VASH1, RCAN1, RBP1,* and *ALDH1A1* could be gene markers to differentiate AF patients and controls.

## Discussion

AF results from multiple factors; thus, its mechanism is complex. Genetic factors, advanced age, hypertension, and diabetes can cause metabolic, electrical, and structural remodeling of the myocardium, eventually leading to AF (7, 8, 19, 20). Aging is an important risk factor for AF, and most patients with AF are elderly (2). The mechanisms and factors that predispose the development, progression, and regression during aging need to be urgently investigated. The study of aging-related factors and the occurrence of AF has an important role in revealing the pathogenesis of AF and providing a theoretical and experimental basis for the early diagnosis and targeted intervention of AF, which is of great importance.

The traditional gene-level analysis focuses more on strong-effect genes. However, it is challenging to find weak-effect genes. The systematic mining idea of WGCNA is a good complement to the analysis of weak-effect genes. WGCNA strengthens the correlations of strongly-correlated genes after power function treatment. In contrast, the correlations of weakly-correlated genes weaken significantly after power function treatment, thus making the network relationships obey an approximate scale-free network distribution (10, 21). Compared with conventional clustering methods, scale-free network distribution is more characteristic of biological data and can effectively restore the role of genes in biological processes. Therefore, constructing a WGCNA network can help identify and screen important modules and hub genes associated with specific clinical traits.

In this study, RNA-seq datasets downloaded from the GEO were analyzed using WGCNA, identified, and clustered into 19 color modules. The correlation between genes and clinical traits was performed for each module. The green module was most correlated with the AF phenotype, and the pink module was most significantly correlated with the SR phenotype. Finally, 16 hub genes were screened from the green module, and 23 hub genes from the pink module. In line with this finding (2, 22–26), we observed that gene functions were mainly enriched in cAMP-mediated signaling, activation of protein kinase A activity, regulation of inflammatory response, and cellular calcium ion homeostasis in the green module. The pink module gene functions were mainly enriched in the ROS metabolic process, Wnt signaling pathway, aging, calcium ion transport, and regulation of mitochondrial membrane potential. In addition, KEGG pathway analysis showed that the green module gene was mainly enriched in the calcium signaling pathway, cAMP signaling pathway, and PPAR signaling pathway, while the pink module gene was mainly enriched in the TGF-*β* signaling pathway, suggesting that the main causes of AF were abnormal calcium homeostasis, energy metabolism, and fibrosis, which is consistent with previous studies (7, 8, 19, 20).

We screened 16 hub genes in the green and 23 in the pink modules and validated this finding in peripheral blood leukocytes from elderly AF patients and controls. RT-qPCR showed that *PTGDS, COLQ, ASTN2, VASH1*, and *RCAN1* were highly expressed in the AF group, whereas *AMIGO2, RBP1, MFAP4,* and *ALDH1A1* were highly expressed in the control group. In addition, ROC analysis indicated that seven of these genes have high diagnostic value as biomarkers for AF.

*PTGDS*, a member of the lipocalin superfamily, plays dual roles in prostaglandin metabolism and lipid transport, which are involved in various cellular processes. *PTGDS* expression is correlated with advanced tumor stages, metastasis, and poor prognosis (27–29). Mallmann et al. have reported that *PTGDS* also regulates voltage-gated CaV2.2 Ca^2+^ channels (30, 31). However, the role of *PTGDS* in AF has not been investigated.

Studies have shown that mutations in *COLQ* can lead to congenital myasthenic syndrome (CMS), which causes cardiac autonomic dysfunction (32, 33). Interestingly, Çubukçuoğlu et al. (34) found that the expression of *COLQ* was higher in degenerative mitral regurgitation patients with AF than in those with SR (*P* = 0.003), which is consistent with the results of our study.

*ASTN2*, a large vertebrate-specific transmembrane protein, is primarily expressed in the developing and adult brain, with the highest levels detected in the cerebellum (35, 36). Behesti et al. (37) demonstrated that *ASTN2* binds to and regulates the surface expression of multiple synaptic proteins in postmigratory neurons by endocytosis, resulting in the modulation of synaptic activity. In addition, by systematic analysis, Burt et al. (38) found that *ASTN2* has pleiotropic effects on cardiometabolic and psychiatric traits. However, *ASTN2* role in AF remains unclear.

*VASH1* is an endothelium-derived negative feedback regulator of angiogenesis (39). *VASH1* is involved in tumorigenesis, atherosclerosis, age-dependent macular degeneration, and diabetic retinopathy (40, 41). Wang et al. (42) reported that the expression of *VASH1* increased rapidly in the ischemic myocardium following AMI. However, the role of *VASH1* in AF has not been explored.

*RCAN1*, previously known as *DSCR1*/*MCIP1*/*Calcipressin*-1/*Adapt78* in mammals, belongs to a family of endogenous regulators of calcineurin activity (43). *RCAN1* has been implicated in maintaining heart function. Mutations in *RCAN1* can lead to congenital heart disease (44). In addition, *RCAN1* contributes to the maintenance of mitochondrial function and modulates tissue damage during myocardial ischemia-reperfusion (45). Xiao et al. (46) recently reported that *RCAN1* might be a novel biomarker for persistent AF.

*AMIGO2*, a novel member of the gene family encoding type I transmembrane proteins, has been studied in cancer research (47). Ma et al. (48) showed that the loss of *AMIGO2* causes dramatic damage to cardiac preservation after ischemic injury. However, the role of *AMIGO2* in AF has not been studied.

*RBP1*, an intracellular chaperone that binds retinol and retinal with high affinity, protects retinoids from non-specific oxidation and delivers retinoids to specific enzymes (49). Yu et al. (50) found that altered *RBP1* expression affects epithelial cell retinoic acid, proliferation, and microenvironment. However, its role in AF remains unclear.

*MFAP4*, also known as *MAGP*-36 in some species, is produced by vascular smooth muscle cells and is highly enriched in the blood vessels of the heart and lungs, contributing to the structure and function of elastic fibers (51, 52). *MFAP4* is involved in cardiac remodeling and its deletion attenuates the progression of angiotensin II-induced atrial fibrosis and AF (53). In addition, *MFAP4* is associated with aneurysms and peripheral arterial diseases (54).

*ALDH1A1*, a member of the *ALDH* family, is highly expressed by stem cells in cancer (55). Da et al. (56) recently found that *ALDH1A1* was associated with cardiac development and protection after MI. However, the role of *ALDH1A1* in AF has not been explored.

In summary, WGCNA analysis showed that the green and pink modules were highly relevant to AF. Hub genes screened from the modules, such as *PTGDS, COLQ, ASTN2, VASH1, RCAN1, AMIGO2, RBP1, MFAP4,* and *ALDH1A1,* may be involved in the occurrence and progression of AF by regulating biological processes, including calcium homeostasis, energy metabolism, fibrosis, inflammatory response, and mitochondrial function. Experiments using samples of patients further validated the differential expression of hub genes screened by WGCNA analysis in different groups and explored their feasibility as biomarkers of AF. This study also identified six novel AF-related genes not previously reported, such as *PTGDS, ASTN2, VASH1, AMIGO2, RBP1*, and *ALDH1A1*, which may be important regulators of AF. However, given the limited samples of vitro experiment in our study, the role of hub genes and mechanisms need to be further identified through in the larger vitro or vivo study(such as heart tissue of AF patients), in order to pave the way for the biomarkers development of AF. In conclusion, our findings provide a theoretical and experimental basis for screening and preventing AF.

## Data Availability

The datasets presented in this study can be found in online repositories. The names of the repository/repositories and accession number(s) can be found in the article/**Supplementary Material**.
