# Supplementary material for: Identification and validation of key genes associated with atrial fibrillation in the elderly
Source: Front Cardiovasc Med. 2023 Mar 29;10:1118686. doi: 10.3389/fcvm.2023.1118686 (PMC10090400; doi:10.3389/fcvm.2023.1118686)
Supplement: Supplementary file 1 [file Table1.docx]

Supplementary Material

# Supplementary Table S1. Clinical characteristics of patients

|  | **dataset GSE2240** | | **patients** | |
| --- | --- | --- | --- | --- |
|  | **SR (n = 20)** | **AF (n = 10)** | **SR (n = 9)** | **AF (n = 9)** |
| **Age (mean ± SD)** | **62.6 ± 10.9** | **62.9 ± 17.9** | **89.6 ± 7.7** | **87.2 ± 6.1** |
| **Gender (female, %)** | **8 (40%)** | **3(30%)** | **2(22.2%)** | **2(22.2%)** |
| **BMI (kg/m^2^)** |  |  | **23.8 ± 2.6** | **25.5 ± 2.1** |
| **SBP(mmHg)** |  |  | **135.7 ± 9.7** | **133.6 ± 14.5** |
| **DBP(mmHg)** |  |  | **70.2 ± 8.1** | **69.3 ± 6.5** |
| **Hypertension(%)** |  |  | **6 (66.7%)** | **4 (44.4%)** |
| **Smoking (%)** |  |  | **3 (33.3%)** | **5 (55.6%)** |
| **DM(%)** | **2 (10%)** | **1 (10%)** | **3 (33.3%)** | **3 (33.3%)** |
| **CAD(%)** | **0 (0%)** | **1 (10%)** | **4 (44.4%)** | **6 (66.7%)** |
| **Hyperlipidemia(%)** |  |  | **4 (44.4%)** | **3 (33.3%)** |
| **Stroke(%)** |  |  | **2 (22.2%)** | **1 (11.1%)** |

**SR = sinus rhythm, AF = atrial fibrillation; BMI = body mass index; DM = diabetes mellitus; CAD = coronary artery disease.**
