# Supplementary material for: Identification and validation of key genes associated with atrial fibrillation in the elderly
Source: Front Cardiovasc Med. 2023 Mar 29;10:1118686. doi: 10.3389/fcvm.2023.1118686 (PMC10090400; doi:10.3389/fcvm.2023.1118686)
Supplement: Supplementary file 2 [file Table2.docx]

Supplementary Material

**Supplementary Table S2. Primer sequences for RT-qPCR**

| **Target name** |  | **Primer** |
| --- | --- | --- |
| **GAPDH** | F | 5’-CCAGGTGGTCTCCTCTGACTTC-3’ |
|  | R | 5’-GTGGTCGTTGAGGGCAATG-3’ |
| **PTGDS** | F | 5’-AGTGGTGGAGACCGACTACGAC-3’ |
|  | R | 5’-ACAATGGTATCCTCTGTGAAGCC-3’ |
| **COLQ** | F | 5’-ACCGTGCCTACTGTGGAGATGG-3’ |
|  | R | 5’-GTCAGGTAGCCAAAGTCAGAGCC-3’ |
| **ASTN2** | F | 5’-ATCTACCTGTCACCTTTGCCGC-3’ |
|  | R | 5’-CCTCCTTTGTGCCATTGTCTTG-3’ |
| **VASH1** | F | 5’-GCTCAAGATTGGCAAAGGGACG-3’ |
|  | R | 5’-GTCTTTCACTGCGGCTGTTCCTG-3’ |
| **RCAN1** | F | 5’-GGAAACAAGTGGAAGATGCGACC-3’ |
|  | R | 5’-TGTCAGTCGCTGCGTGCAATTC-3’ |
| **AMIGO2** | F | 5’-TTTTCTCACACAGTTTCCGATGG-3’ |
|  | R | 5’-GATACCAAAAGACCAGCAAGGAG-3’ |
| **RBP1** | F | 5’-ACTTGCTGAAGCCAGACAAAGAGAT-3’ |
|  | R | 5’-CCTCAAACTCCTTCCCAACCTG-3’ |
| **MFAP4** | F | 5’-GGCTCAGTAAGTTTCTTCCGCG-3’ |
|  | R | 5’-CCAAGTCCACTCGCAGCTCATA-3’ |
| **ALDH1A1** | F | 5’-GCCAGGTAGAAGAAGGAGATAAGG-3’ |
|  | R | 5’-CCCTCTCGGAAGCATCCATAG-3’ |
